# Retraction: *In silico* elucidation of potential drug targets against oxygenase domain of Human eNOS Dysfunction

**DOI:** 10.1371/journal.pone.0362427

**Published:** 2026-11-10

**Authors:** The PLOS One Editors

The *PLOS One* Editors retract this article [1] due to concerns about:

- Compliance with PLOS policies on Authorship.
- Compromised peer review.

We regret that the issues were not identified prior to the article’s publication.

MNI and SAS did not agree with the retraction. AM, SA, MSK, SS, MFS, ZS, and MS either did not respond directly or could not be reached.

## References

[1] Malik A, Iqbal MN, Ashraf S, Khan MS, Shahzadi S, Shafique MF, et al. RETRACTED: *In silico* elucidation of potential drug targets against oxygenase domain of Human eNOS Dysfunction. PLoS One. 2023;18(4):e0284993. doi: 10.1371/journal.pone.0284993 37099543 PMC10132633

